# Distinct physiological responses of tomato and cucumber plants in silicon-mediated alleviation of cadmium stress

**DOI:** 10.3389/fpls.2015.00453

**Published:** 2015-06-16

**Authors:** Jiawen Wu, Jia Guo, Yanhong Hu, Haijun Gong

**Affiliations:** College of Horticulture, Northwest A&F UniversityYangling, Shaanxi, China

**Keywords:** antioxidant defense, cadmium, Cd transport, cell wall polysaccharide, organic acid, silicon

## Abstract

The alleviative effects of silicon (Si) on cadmium (Cd) toxicity were investigated in cucumber (*Cucumis sativus* L.) and tomato (*Solanum lycopersicum* L.) grown hydroponically. The growth of both plant species was inhibited by 100 μM Cd, but Si application counteracted the adverse effects on growth. Si application significantly decreased the Cd concentrations in shoots of both species and roots of cucumber. The root-to-shoot transport of Cd was depressed by added Si in tomato whereas it was increased by added Si in cucumber. The total content of organic acids was decreased in tomato leaves but increased in cucumber roots and leaves by Si application under Cd stress. Si application also increased the cell wall polysaccharide levels in the roots of both species under Cd toxicity. Si-mediated changes in levels of organic acids and cell wall polysaccharides might contribute to the differences in Cd transport in the two species. In addition, Si application also mitigated Cd-induced oxidative damage in both species. The results indicate that there were different mechanisms for Si-mediated decrease in shoot Cd accumulation: in tomato, Si supply decreased root-to-shoot Cd transport; whereas in cucumber, Si supply reduced the Cd uptake by roots. It is suggested that Si-mediated Cd tolerance is associated with different physiological responses in tomato and cucumber plants.

## Introduction

In recent decades, extensive industrial and anthropogenic activities have caused serious heavy metal pollution. As a highly mobile and toxic element, cadmium (Cd) pollution is increasingly of concern. Cd toxicity can disturb morphological, biochemical and ultrastructural functions in plants (Ali et al., 2013, 2014). Although Cd does not participate in Fenton-type reaction directly, it can indirectly induce production of reactive oxygen species (ROS) such as superoxide radical (O_2_^-^), hydrogen peroxide (H_2_O_2_), and hydroxyl radical (●OH) (Stohs and Bagchi, 1995; Gonçalves et al., 2007). Excessive ROS production can disturb the balance of nutritional status and induce oxidative damage. What is more, Cd accumulation in crops and vegetables that exceeds the safety threshold will endanger human health via food chain (Aery and Rana, 2003; López-Millán et al., 2009).

Silicon (Si) is regarded as a beneficial element that is taken up by plant roots as silicic acid (Epstein, 1999). According to the modes of Si uptake, plants can be classified into three groups (active, passive, and exclusive uptake), and the corresponding plants are high, intermediate and low Si accumulators: examples are rice (*Oryza sativa* L.), cucumber (*Cucumis sativus* L.), and tomato (*Solanum lycopersicum* L.), respectively (Mitani and Ma, 2005). Many studies have shown that Si can alleviate heavy-metal-induced adverse effects on plant growth (Shi et al., 2005; Dragisic Maksimovic et al., 2012; Vaculík et al., 2012). It has been reported that, as a modulator of stress tolerance, Si exerts its beneficial roles mainly through two mechanisms: the protective effects of amorphous silica as a physical barrier, and the biochemical functions of aqueous silicic acid (Ma and Yamaji, 2008; Cooke and Leishman, 2011). The mechanism by which Si acts as a physical barrier has been well documented (Ma and Yamaji, 2008), whereas there is much less evidence for the direct biochemical function of Si in plants. Under Cd toxicity, Si application has been found to alleviate oxidative stress and affect root structure (Liang et al., 2005; Shi et al., 2005; Nwugo and Huerta, 2008, 2011; da Cunha and do Nascimento, 2009; Song et al., 2009; Liu et al., 2013b). However, the mechanism for Si-mediated Cd tolerance is still not fully understood, and the majority of previous studies have focused on cereal plants such as maize (*Zea mays* L.) and rice, which are high Si accumulators (Liang et al., 2005; Shi et al., 2005; Nwugo and Huerta, 2008, 2011; da Cunha and do Nascimento, 2009; Lukačová Kuliková and Lux, 2010; Liu et al., 2013a), where a physical blockage by Si deposition in these plants may have contributed to the decreased shoot Cd accumulation and therefore enhanced Cd tolerance (Shi et al., 2005). Up to date, relatively few papers have reported Si-alleviated Cd toxicity in vegetables, which are usually low Si accumulators (Shi et al., 2010; Farooq et al., 2013). Recently, Katz (2014) also pointed out the importance of studying Si accumulation and its function in non-grass species, with low capabilities of Si accumulation. In addition, the changes of Cd accumulation and translocation in plants due to Si addition reported in previous studies have been mixed. For example, previously, Song et al. (2009) found that Si addition decreased Cd uptake and transport in pakchoi. In maize, Vaculík et al. (2009) observed that addition of Si did not decrease the Cd concentration in the shoot and it even slightly increased the shoot Cd concentration at low Cd level. Therefore, the regulative role of Si on Cd uptake and transport in different plants still needs to be investigated, and investigation of the role of Si in less-Si-accumulating plants will help clarify the biochemical mechanism(s) of Si-mediated stress tolerance.

Cucumber and tomato are two important vegetables that suffer from Cd toxicity in areas with heavy-metal pollution (López-Millán et al., 2009; Feng et al., 2010). Reducing Cd accumulation in these vegetables is urgent to guarantee food safety. Compared with cereal crops, cucumber and tomato plants accumulate less Si. Using these low Si-accumulating plants as models should help clarify the possible biochemical mechanism of Si-mediated Cd tolerance in plants. However, to our knowledge, little information is available on the effects of Si on Cd toxicity in cucumber and tomato. Feng et al. (2010) found that Si could alleviate Cd-induced retardation of photosynthesis and nitrogen metabolism in cucumber, but Si-mediated alleviation of Cd toxicity in relation to Cd distribution and antioxidant defense remains to be investigated. In tomato, relevant information is lacking.

Organic acids can bind with heavy metals to form metal-organic acid complexes, which are involved in root-to-shoot transport of the metals in plants (Senden et al., 1995; Nigam et al., 2000; Han et al., 2006; Lu et al., 2013). Previous studies have shown that the plant organic acids concentrations are differently influenced by Cd toxicity (Boominathan and Doran, 2003; Tian et al., 2011; Xu et al., 2012; Lu et al., 2013). However, it remains unknown whether Si affects organic acid contents in a way that influences heavy metal transport in plants. On the other hand, the cell wall is the first barrier for stress defense in roots, and the number of functional groups (-OH, -COOH, and -SH) in the cell wall components determines the capability of binding metal cations (Krzeslowska, 2011). Binding metal ions with the cell wall constituents can confine toxic metal to the walls and decrease their concentrations in the symplast (Krzeslowska, 2011; Yang et al., 2011). Liu et al. (2013a) observed Cd–Si co-complexation in the cell-wall matrix in rice suspension cells and protoplasts. Ma et al. (2015) further reported that the formation of [Si-hemicellulose matrix]Cd complexation in the rice cell walls contributed to the inhibition of Cd uptake. However, little information is available on the possible modulation of Si on the levels of cell wall constituents (such as pectin, hemicellulose, cellulose and lignin) under Cd toxicity.

Plants have evolved a complex defense system against ROS, consisting of enzymatic antioxidants such as superoxide dismutase (SOD), catalase (CAT), ascorbate peroxidase (APX) and glutathione reductase (GR), and non-enzymatic constituents such as non-protein thiol, ascorbic acid (AsA), glutathione (GSH) (Tiryakioglu et al., 2006). SOD can convert O_2_^-^ to H_2_O_2_ and O_2_ while CAT is able to decompose H_2_O_2_ to H_2_O and O_2_ (Balakhnina and Borkowska, 2013). As key enzymes in ascorbate-glutathione cycle, APX and GR play important roles in scavenging excessive H_2_O_2_ in plants (Rennenberg, 1980). It has been observed that Si could alleviate Cd toxicity by enhancing antioxidant defense (Liu et al., 2013b). However, the possible role of antioxidant defense in Si-mediated alleviation of Cd toxicity in either tomato or cucumber has not been systematically investigated.

In this study, the effects of Si on Cd uptake, translocation and distribution, antioxidant defense, organic acids and cell wall components in cucumber and tomato under Cd stress were investigated, with the aim being to clarify the mechanisms for Si-mediated Cd tolerance in these plants contrasting in Si-accumulating capabilities.

## Materials and Methods

### Plant Materials and Treatments

Seeds of tomato (*S. lycopersicum* L. cv. Jinpengchaoguan) and cucumber (*C. sativus* L. cv. Xinyan No.7) were surface-sterilized with 10% H_2_O_2_ for 15 min and germinated for 2 days at 25°C (tomato) or 28°C (cucumber) in the dark. Germinated seeds were transferred to a growth chamber (25/15°C, day/night; 14/10 h, light/dark, and relative humidity of 70%) and uniform seedlings transplanted to 10-L plastic pots when the third leaf was expanding.

The seedlings were grown in half-strength Hoagland solution which contained macro elements (mM): KNO_3_ 2.5, Ca(NO_3_)_2_⋅4H_2_O 2.5, MgSO_4_⋅7H_2_O 1, KH_2_PO_4_ 0.5; and micro elements (μM): H_3_BO_3_ 46.1, MnCl_2_⋅4H_2_O 9.1, ZnSO_4_⋅7H_2_O 0.76, CuSO_4_⋅5H_2_O 0.32, Na_2_MoO_4_⋅2H_2_O 0.24. Iron was provided as EDTA-Fe at 50 μM. Before Cd stress, half of plants were pretreated for 2 weeks with Si as Na_2_SiO_3_⋅9H_2_O (0.5 and 2 mM for cucumber and tomato, respectively). The solution pH was adjusted to 6.0 with NaOH or HCl solution. Additional NaCl was added to treatments without Si application to compensate the Na^+^ introduced by Si supply. After Si pretreatment, the plants were subjected to 100 μM CdCl_2_ treatment without or with added Si. There were four treatments: control (CT, without Si or Cd treatment), Si, Cd, and Si + Cd. The nutrient solution was renewed every 3 days with continuous supply of air. After 15 days of Cd exposure, the plants were harvested for determinations.

### Determination of Cd Concentration

Plants were divided into shoots and roots. The roots were washed with distilled water at least three times, and were then immersed in ice-cold 0.5 mM CaCl_2_ for 20 min, after which they were dried at 70°C for 3 days. Dry plant materials were ground to powder, weighed, and digested in mixed nitric acid-perchloric acid (v:v, 4:1) solution (Liu et al., 2013b). The Cd concentrations in digested solutions were determinate by atomic adsorption spectrophotometry (Hitachi Z-2000, Japan).

Root-to-shoot translocation factor (*TF*) of Cd was calculated as follows:

TF = [Cd]_shoot_/[Cd]_root_ × 100% (Shi et al., 2010)

### Fractionated Extraction of Cd

Roots and laminae were fractionated according to Dragisic Maksimovic et al. (2012) with slight modifications. Briefly, tissues were infiltrated with 0.4 M sucrose, then frozen with liquid nitrogen and homogenized first with 0.4 M sucrose and then with 1% (w/v) SDS. Each homogenate (sucrose and sucrose followed by SDS) was washed three times with the corresponding solutions (sucrose or SDS) and three times with distilled water. Cd extracted by sucrose represented weakly-bound symplastic Cd (sucrose extractable Cd) while extraction by SDS was symplastic Cd bound to the proteins (protein extractable Cd), and the pellet was cell wall (cell wall bound Cd).

### Si Concentration Determination

Silicon in tissues was extracted with hydrochloric acid and hydrofluoric acid according to Heine et al. (2005) and Si concentrations were determined based on Dai et al. (2005). Briefly, the powders of dried tissues were incubated in a mixture of 1 M hydrochloric acid and 2.3 M hydrofluoric acid (v:v, 1:2) for 24 h. After incubation, the solutions were centrifuged and the supernatants were further incubated with 3.2% (w/v) boric acid for another 24 h. Aliquots of 10 mL ammonium molybdate tetrahydrate (54 g L^-1^, pH 7.0) was added into 1 mL of the supernatant. Five minutes later, 1 mL of reducing reagent consisted of solution A (0.2 g Na_2_SO_3_ and 0.4 g 1-amino-2-naphthol-4-sulfonic acid in 25 mL of distilled water) and solution B (25 g NaHSO_3_ in 200 mL of distilled water) and 5 mL 20% (w/v) tartaric acid were rapidly added. After 30 min reaction, the supernatant were measured at absorbance at 650 nm.

### Determination of Organic Acid Contents

Tissues were extracted in 0.5 M HCl at 60°C for 1 h, and then centrifuged at 8,000 *g* for 15 min. After that, the supernatant was filtered through a 0.22 μM millipore filter before organic acid analysis by high-performance liquid chromatography (HPLC, Waters, 1525, America). The mobile phase was 20 mM KH_2_PO_4_ (pH 2.25) at a flow rate of 0.8 mL min^-1^, and the total run time was 35 min. Organic acids were monitored at 210 nm. The injection volume was 10 μL and the concentration was calculated based on peak area (Xu et al., 2012).

### Root Cell Wall Isolation and Analysis

Root tissues (about 3 g) were ground into powder with liquid nitrogen, and the powder then immersed into 30 mL of cold ethanol. After 20 min, the solution was centrifuged at 1,000 *g* for 20 min, and the supernatant discarded. The pellet was washed with 20 mL of cold acetone, 20 mL of a mixture of methanol and chloroform (1:1; v/v) and 20 mL of methanol successively, and the wash was conducted at least twice with each solution. The final pellet was dried with a freeze-dried (CS110-4, Scanvac, Denmark), and the dried pellet was regarded as cell wall (Zhong and Lauchli, 1993).

The constituents of cell wall were analyzed by fourier transform infrared spectroscopy analyzer (FTIR; Vetex 70, Bruker, Germany). Spectra were recorded from 1800 to 900 cm^-1^ with a resolution of 0.4 cm^-1^. Before detection, every sample was normalized with background scanning. The cell wall materials were mixed with dried KBr (1: 100) and ground with agate mortar and pestle, then the mixture was weighed and pressed with a tablet machine (no more than 10 KPa). The tablet was scanned with the FTIR (Regvar et al., 2013).

### 8-Hydroxy-1, 3, 6-Pyrenetrisulphonic Acid (PTS) Uptake

An apoplastic tracer, 8-hydroxy-1, 3, 6-pyrenetrisulphonic acid (abbreviated as PTS), was used to determine the apoplastic transport of Cd. Following Si pretreatment, the plants were subjected to 100 μM Cd treatment in growth solution without or with 30 mg L^-1^ PTS for 24 h. The shoots of six plants without PTS treatment and 12 plants with PTS treatment were harvested for fluorescence analysis. The shoots were extracted in deionized water at 90°C for 2 h. PTS fluorescence was determined with an excitation wavelength of 403 nm and emission wavelength of 510 nm according to Gong et al. (2006) with a fluorescence spectrophotometer (Hitachi F-4600).

### Determination of TBARS and Hydrogen Peroxide (H_2_O_2_) Contents

Plant tissues (0.5 g) were homogenized in ice-cold 5% (w/v) trichloroacetic acid (TCA) solution. After centrifugation at 3000 *g* for 10 min, the supernatant was used to determine thiobarbituric acid reactive substances (TBARS) using the method of Heath and Packer (1968).

H_2_O_2_ contents were assayed according to Velikova et al. (2000). Potassium iodide (KI) was applied to detect H_2_O_2_ contents at an absorbance of 390 nm.

### Determination of Membrane Stability Index (MSI)

The third-oldest fresh leaves of tomato and cucumber seedlings were washed with deionized water and cut into leaf discs using a hole punch. Twenty discs were put into one clean glass tube with 10 mL of deionized water and evacuated for 10 min during which the tubes were shaken every 2 min. Fresh roots (0.2 g) were weighed and also transferred into clean glass tube with 10 mL deionized water. Every treatment had two sets, and each set had four replicates. One set of the tubes with leaf discs or roots were heated at 40°C for 30 min, and another set was heated at 100°C for 10 min. After the solution cooled, its electrical conductivity was recorded (DDS-307 conductivity meter, Shanghai, China). The MSI was calculated as follows (Rodriguez-Hernandez et al., 2013):

MSI = (1 – C1/C2) × 100%, where C1 and C2 represented the electrical conductivities of solutions in which the tissues were heated at 40°C and 100°C, respectively.

### Antioxidant Enzyme Extraction and Activity Assays

Plant tissues were homogenized in ice-cold 50 mM potassium phosphate buffer solution (pH 7.8) containing 0.2 mM EDTA-Na_2_. After centrifugation at 12,000 *g* for 20 min at 4°C, the supernatant was used to determine enzyme activities. Soluble protein contents were assayed by the method of Bradford (1976) using bovine serum albumin as a standard.

Superoxide dismutase and CAT activities were determined according to Dhindsa et al. (1981). Nitroblue tetrazolium (NBT) method was used for determination of SOD activity and one unit SOD activity was defined as the amount of enzyme required to cause a 50% inhibition of the rate of NBT reduction. The reaction mixture for CAT contained 25 mM potassium phosphate buffer and 10 mM H_2_O_2_, and the reaction was initiated by addition of enzyme extract: the absorbance was determined at 240 nm after 180 s.

Ascorbate peroxidase activity was measured based on the decrease of AsA content at 290 nm because AsA was oxidized by APX (Nakano and Asada, 1981). Determination of GR activity was based on the decrease of absorbance at 340 nm as NADPH was oxidized to NADP in the presence of GR enzyme (Fryer et al., 2002).

### Determination of Reduced Glutathione (GSH) and Ascorbic Acid Contents

Glutathione content in plant tissues was determined according to Guri (1983) with slight modifications. Fresh tissue (1 g) was homogenized in 5 mL of 3% (w/v) TCA containing 5 mM EDTA in an ice bath, and the homogenate centrifuged at 4,000 *g* for 10 min. Aliquots of 1 mL of the supernatant were titrated with about 0.2 mL of 1 M NaOH to adjust the pH to 6.5–7.0. Then 0.5 mL of 50 mM K-phosphate buffer (pH 7.0) and 0.05 mL of 1 mM dithiobis-2-nitrobenzoic acid (DTNB) were added. After 5 min at room temperature, the absorbance at 412 nm was recorded. The GSH concentration was calculated using a standard curve.

Reduced AsA content in plant tissues was measured according to Arakawa et al. (1981) with small modifications. One gram of fresh tissue was homogenized in 5 mL of 5% (w/v) TCA in an ice bath, and the homogenate centrifuged at 4,000 *g* for 10 min. To 1 mL of the supernatant was added 1 mL of 5% (w/v) TCA, 1 mL ethanol, 0.5 mL of 0.4% (v/v) H_3_PO_4_-ethanol, 1 mL of 0.5% (w/v) bathophenanthroline (BP)-ethanol, and 0.5 mL of 0.03% (w/v) FeCl_3_-ethanol. The mixture was incubated at 30°C for 90 min, and then the absorbance at 534 nm was recorded. The AsA content was determined using a standard curve.

### Statistical Analysis

The data were analyzed by One-Way ANOVA or *t*-test. Comparison of data was subjected to LSD test at a significant level of 0.05 using SPSS software (SPSS Inc. 16.0).

## Results

### Plant Growth

**FIGURE 1:**
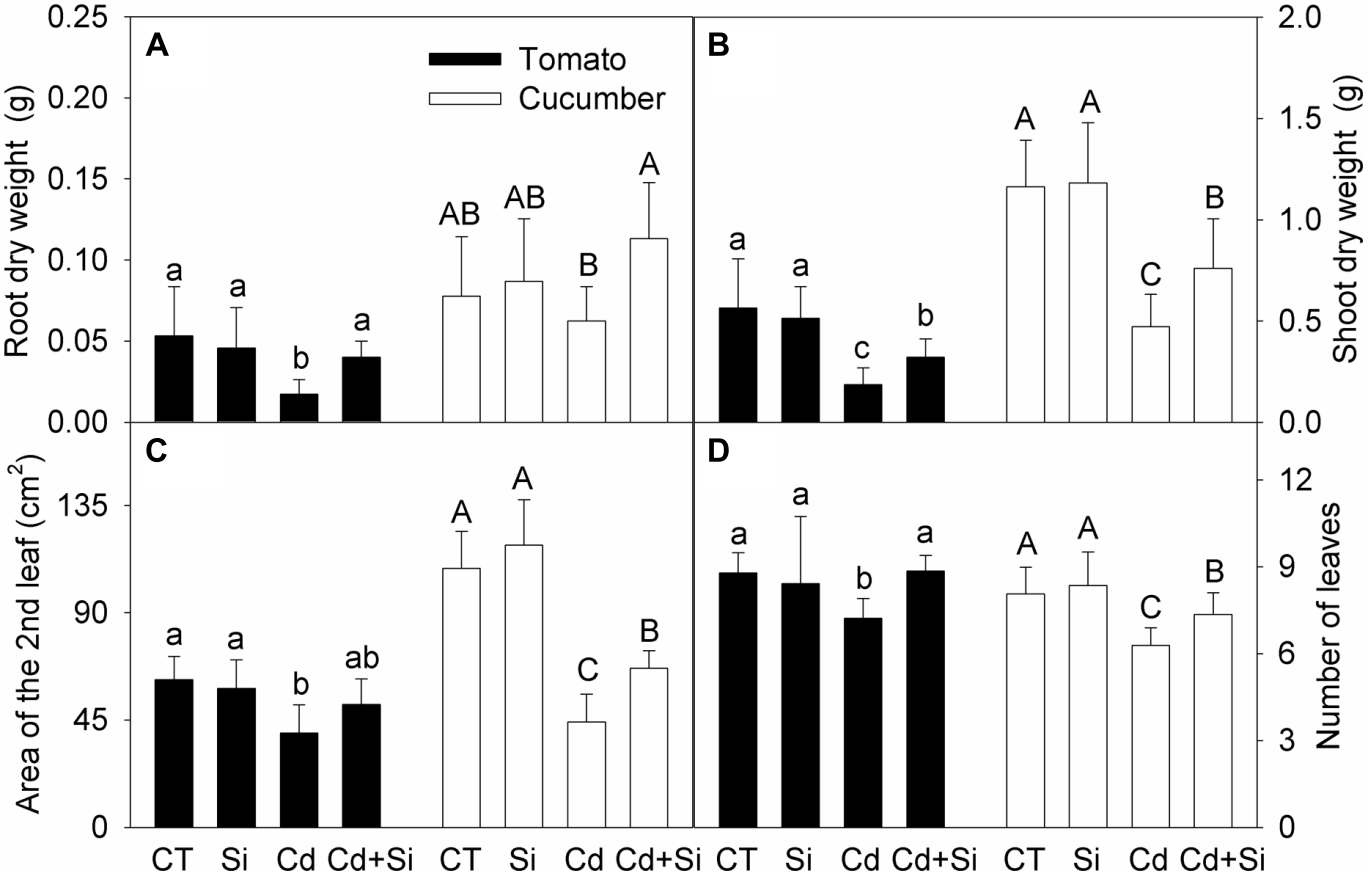
**Effects of silicon (Si) on dry weights of root (A) and shoot (B), the second leaf area (C) and total number of leaves (D) in tomato and cucumber plants under cadmium (Cd) stress**. Bars with different letters means significant difference at *P* < 0.05 according to one-way ANOVA. Lower-case and upper-case letters indicate significance for tomato and cucumber, respectively.

Under non-stress conditions, Si addition had no obvious effect on the growth of tomato and cucumber plants (**Figures 1A–D**). Cd treatment significantly decreased the dry mass, leaf area and number in both tomato and cucumber (except the root dry weight in cucumber; **Figures 1A–D**). Under Cd toxicity, Si supply significantly increased these parameters (except the second leaf area of tomato, **Figure 1C**) and improved plant growth.

### Si Accumulation

From our preliminary experiments, the optimum Si levels for Cd stress alleviation were 2 and 0.5 mM for tomato and cucumber, respectively. Si concentrations in the shoot and roots were 2–3 times higher in Si-applied plants than in the corresponding controls (**Table 1**). Although the Si level for treating cucumber was only one fourth of that for tomato, the shoot Si concentrations were even higher in cucumber than in tomato (**Table 1**).

**Table 1:** Silicon (Si) concentrations in tomato and cucumber as affected by Cd and Si.

	Si concentration (μg g^-1^ DW)			
Treatment	Tomato		Cucumber	
	Shoot	Root	Shoot	Root
CT	710 ± 219^b^	1170 ± 292^b^	1447 ± 456^b^	1621 ± 378^b^
Si	2324 ± 624^a^	4005 ± 643^a^	3520 ± 552^a^	3315 ± 604^a^
Cd	803 ± 108b	1922 ± 543^b^	1071 ± 114^b^	1440 ± 104^b^
Si + Cd	2023 ± 358^a^	4099 ± 655^a^	3264 ± 647^a^	3721 ± 700^a^

*Data are means ± SD. Values followed by different letters within a column represent significant difference at P< 0.05 according to one-way ANOVA*.

### Cd Accumulation and Subcellular Distribution

The root-to-shoot Cd translocation was significantly higher in tomato than in cucumber (**Table 2**). Si application significantly decreased the shoot Cd concentrations in both tomato and cucumber (**Table 2**) and the root Cd concentration in cucumber. The root-to-shoot Cd *TF* was decreased by added Si in tomato, but increased in cucumber (**Table 2**).

**Table 2:** Cadmium concentrations and translocation factors (*TF*) in tomato and cucumber grown hydroponically without or with added Si.

	Cd concentration (μg g^-1^ DW)				TF (%)	
	Tomato		Cucumber		Cd_shoot_/Cd_root_	
Treatment	Shoot	Root	Shoot	Root	Tomato	Cucumber
Cd	1136 ± 125^a^	6931 ± 893^a^	659 ± 35.3^a^	13346 ± 2069^a^	16.8 ± 2.5^a^	4.9 ± 0.4^b^
Si + Cd	799 ± 54.8^b^	6232 ± 353^a^	550 ± 24.2^b^	9486 ± 1219^b^	13.0 ± 0.5^b^	5.9 ± 0.3^a^

*Data are means ± SD. Values followed by different letters within a column represent significant difference at *P* < 0.05 according to t-test. Cd concentrations in control and Si treatments were undetected*.

In tomato, Si addition reduced the symplastic Cd in the leaves, whereas in the roots, it only decreased the sucrose extractable Cd concentration. In cucumber, Si supply decreased the concentrations of sucrose extractable and cell wall bound Cd in the leaves, and it decreased the Cd concentrations in all fractions in the roots (**Table 3**).

**Table 3:** Cadmium concentrations in different fractions extracted from the roots and leaves in tomato and cucumber plants.

Species	Tissue	Treatment	Sucrose extractable	Protein bound	Cell wall bound	Total amount	Percentage of cell
			(μg g^-1^ FW)	(μg g^-1^ FW)	(μg g^-1^ FW)	(μg g^-1^ FW)	wall bound (%)
Tomato	Leaf	Cd	36.2 ± 1.3^a^	19.5 ± 0.7^a^	8.1 ± 0.7^a^	63.8 ± 2.1^a^	12.7
		Si + Cd	27.9 ± 2.3^b^	10.6 ± 0.4^b^	7.7 ± 1.2^a^	46.3 ± 3.2^b^	16.6
	Root	Cd	334.4 ± 21.0^a^	18.4 ± 0.4^a^	262.8 ± 40.6^a^	615.7 ± 60.7^a^	42.7
		Si + Cd	269.4 ± 8.7^b^	15.1 ± 2.7^a^	242.1 ± 30.5^a^	526.6 ± 47.4^a^	46.0
Cucumber	Leaf	Cd	20.8 ± 2.0^a^	6.6 ± 0.5^a^	12.5 ± 0.8^a^	39.9 ± 3.9^a^	31.3
		Si + Cd	14.1 ± 0.3^b^	6.3 ± 0.5^a^	7.0 ± 1.1^b^	27.3 ± 1.5b	25.6
	Root	Cd	535.3 ± 20.1^a^	53.9 ± 2.4^a^	334.2 ± 50.6^a^	923.4 ± 38.1^a^	36.2
		Si + Cd	469.4 ± 26.7^b^	38.2 ± 3.1^b^	217.3 ± 13.8^b^	724.8 ± 23.6^b^	30.0

*Data are means ± SD. Values followed by different letters represent significant difference between Cd and Si + Cd treatments within a species and tissue at P< 0.05 according to t-test. Cd concentrations in control and Si treatments were undetected*.

### Organic Acids Levels

Five organic acids were detected in tomato and cucumber plants, with tartaric acid being the main organic acid in both plants (**Tables 4 and 5**).

**Table 4:** Effect of Si on organic acids concentrations in tomato seedlings under Cd toxicity.

Organic acid concentration (μmol g^-1^ FW)							
	
Tissue	Treatment	Oxalic acid	Acetic acid	Tartaric acid	Citric acid	Malic acid	Total
Root	CT	5.98 ± 0.16b	21.78 ± 0.81^d^	722.9 ± 10.4^ab^	3.94 ± 0.18^b^	0.63 ± 0.04^c^	755.3 ± 11.0^a^
	Si	7.66 ± 0.23^a^	28.48 ± 0.97^c^	694.8 ± 5.91^b^	4.25 ± 0.33^b^	3.35 ± 0.27^b^	738.5 ± 7.1^a^
	Cd	7.99 ± 0.75^a^	39.04 ± 1.70^b^	686.9 ± 33.2^b^	5.42 ± 0.20^a^	11.11 ± 1.23^a^	750.5 ± 32^a^
	Si + Cd	7.63 ± 0.32^a^	43.31 ± 2.22^a^	746.2 ± 11.0^a^	2.55 ± 0.27^c^	1.24 ± 0.15^c^	800.9 ± 8.6^a^
Leaf	CT	35.47 ± 0.40^a^	29.17 ± 0.09^b^	1207 ± 76^a^	7.53 ± 0.27^b^	16.75 ± 0.57^c^	1296 ± 76.1^a^
	Si	34.61 ± 0.43^b^	22.84 ± 0.74^c^	1017 ± 32^b^	11.78 ± 1.34^a^	16.19 ± 0.62^c^	1102 ± 31.9^b^
	Cd	34.46 ± 0.47^b^	36.88 ± 0.68^a^	965.7 ± 4.88^b^	12.66 ± 0.13^a^	31.09 ± 0.85^a^	1080 ± 3.34^b^
	Si + Cd	35.27 ± 0.37^ab^	27.96 ± 2.66^b^	760.5 ± 27.2^c^	13.26 ± 1.23^a^	23.18 ± 0.84^b^	860.1 ± 28.7^c^

*Data are means ± SD. Values followed by different letters within a column in a tissue represent significant difference among treatments at P< 0.05 according to one-way ANOVA*.

**Table 5:** Effect of Si on organic acids concentrations in cucumber seedlings under Cd toxicity.

	Organic acid concentration (μmol g^-1^ FW)							
	
Tissue	Treatment	Oxalic acid	Acetic acid	Tartaric acid	Citric acid	Malic acid	Total
Root	CT	1.00 ± 0.05^c^	17.11 ± 0.38^c^	1058 ± 35^a^	1.54 ± 0.24^c^	1.51 ± 0.33	1079 ± 35.5^a^
	Si	0.92 ± 0.01^d^	37.14 ± 2.58^a^	922.1 ± 11.6^b^	1.19 ± 0.10^c^	UD	961.3 ± 10.8^b^
	Cd	1.13 ± 0.03^b^	17.10 ± 0.36^c^	608.5 ± 10.3^d^	4.76 ± 0.28^b^	2.47 ± 0.39	634.1 ± 9.89^d^
	Si + Cd	2.12 ± 0.05^a^	27.25 ± 0.92^b^	688.4 ± 11.6^c^	5.71 ± 0.38^a^	2.73 ± 0.14	726.2 ± 10.7^c^
Leaf	CT	ND	17.72 ± 1.24^b^	1388 ± 9.2^b^	5.29 ± 0.08^ab^	7.13 ± 0.32^c^	1418 ± 19.6^b^
	Si	ND	16.15 ± 1.44b	1461 ± 25.8^a^	5.09 ± 0.58^a^	18.85 ± 0.68^a^	1503 ± 8.14^a^
	Cd	2.91 ± 0.23	22.71 ± 0.59^a^	707.4 ± 6.34^d^	4.79 ± 0.46^b^	13.88 ± 0.74^b^	751.7 ± 9.54^d^
	Si + Cd	2.33 ± 0.14	22.57 ± 2.26^a^	805.8 ± 6.83^c^	4.83 ± 0.16^b^	14.32 ± 0.36^b^	849.8 ± 6.27^c^

*Data are means ± SD. Values followed by different letters within a column in a tissue represent significant difference among treatments at *P* < 0.05 according to one-way ANOVA. ND, not detected*.

In tomato roots, compared with the control, the tartaric acid concentration in Cd-stressed plants was not significantly changed, while the levels of the other four organic acids (especially malic acid) were considerably increased (**Table 4**). Under Cd stress, Si application enhanced the accumulation of tartaric acid and acetic acid while it decreased the levels of malic acid and citric acid. As a result, Si addition did not obviously change the total organic acids level under Cd stress (**Table 4**).

In tomato leaves, the tartaric acid concentration was significantly decreased under Cd stress, while the levels of other organic acids (except oxalic acid) were increased (**Table 4**). However, Si addition decreased the concentrations of tartaric acid, acetic acid, and malic acid in Cd-stressed plants. The concentrations of total organic acids were decreased under Cd stress, and they were further decreased by added Si in stressed plants (**Table 4**).

In cucumber roots, Cd toxicity induced a decrease in tartaric acids concentration and accumulations of malic acid and citric acid (**Table 5**). Under Cd stress, Si-treated plants had higher concentrations of tartaric acid, acetic acid, oxalic acid, and citric acid. The total organic acids level was increased by Si addition under Cd stress (**Table 5**).

In cucumber leaves, the tartaric acid concentration was dramatically decreased, while the levels of acetic acid, oxalic acid, and malic acid were increased under Cd stress (**Table 5**). Under Cd stress, Si supplement significantly increased the tartaric acid level, but it did not affect the contents of other organic acids (**Table 5**).

### FTIR Spectra of Root Cell Walls

**FIGURE 2:**
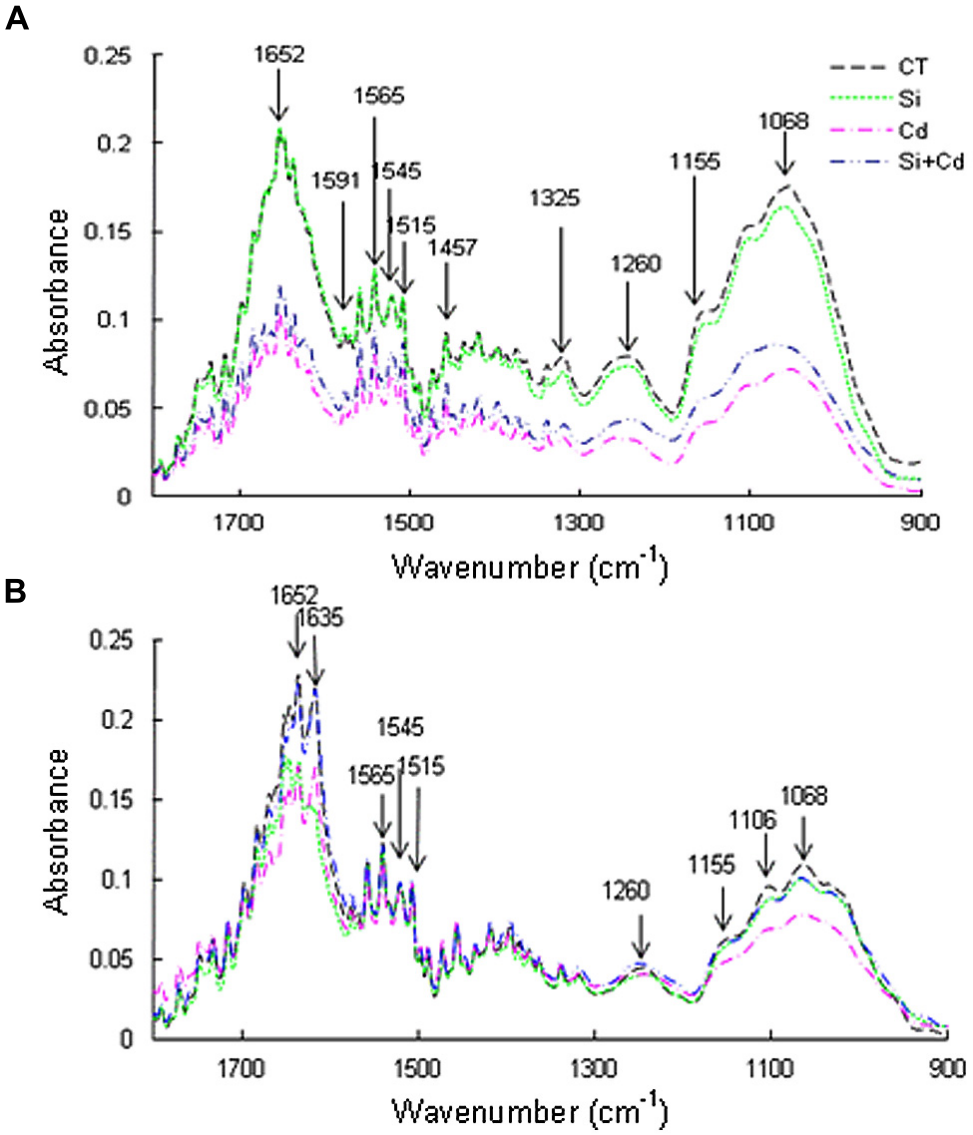
**Effects of Si on changes in fourier transform infrared spectra of root cell walls in tomato (A) and cucumber (B) under Cd stress**.

The spectral peak intensities were higher in CT and Si treatments than in Cd and Si + Cd treatments in both species (**Figure 2**). Under Cd toxicity, the following main peaks were decreased: at 1652 cm^-1^ representing amide I band from protein, at 1605–1590 cm^-1^ indicating carboxyl/acidic groups from pectin or aromatic systems (such as lignin, nitro groups); at 1545 cm^-1^ for stretching vibration C-N and C-H and/or bending vibration N-H in protein (amide I band); at 1515 cm^-1^ for stretching C = C, C = O and ring from lignin; at 1460–1400 cm^-1^ for alicyclic and aliphatic groups from lignin and/or various polysaccharides and carboxyl groups from pectin; at 1325 cm^-1^ for carboxyl groups from cellulosic compounds, ligands and protein; at 1300–1260 cm^-1^ for carboxyl groups from lignin, pectin, and various polysaccharides. The spectra between 900 and 1200 cm^-1^ represent the fingerprints of polysaccharides (Regvar et al., 2013; and references therein). In tomato roots, the spectral peak intensities were decreased under Cd stress, and the absorbance values were obviously higher in Si + Cd treatment than Cd treatment, especially between 900 to 1300 cm^-1^ (**Figure 2A**). In cucumber roots, the absorbance values of spectra of the control, Si and Si + Cd treatments were similar, while those of Cd treatment were obviously lower, especially at 1155, 1105, and 1068 cm^-1^, which indicates cellulosic compounds, pectin and hemicellulose, respectively (**Figure 2B**).

### Apoplastic Transport of Cd

**FIGURE 3:**
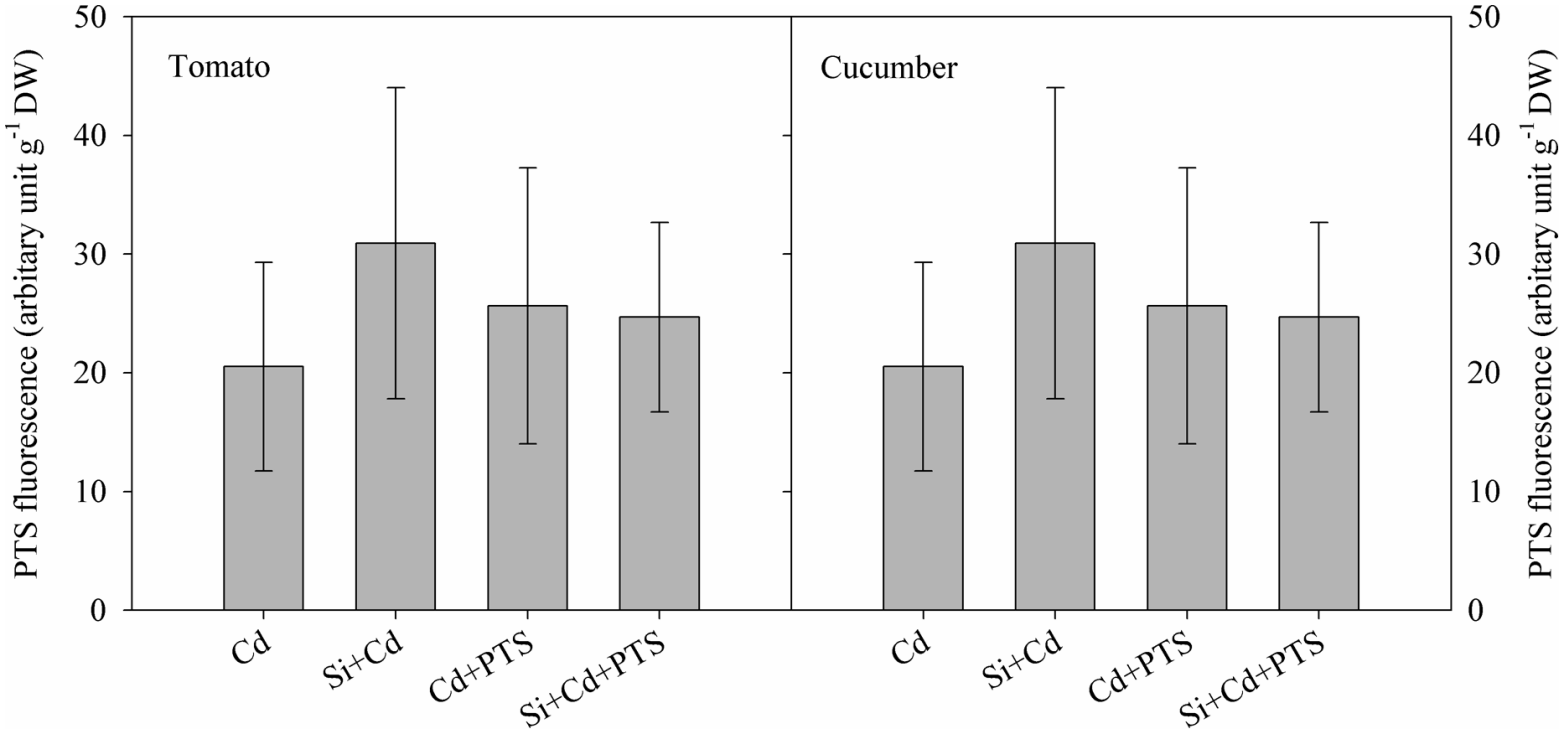
**Fluorescence of leaves as affected by Cd and PTS treatments**.

8-hydroxy-1, 3, 6-pyrenetrisulphonic acid is a membrane-impermeable fluorescent dye, and it has been frequently used as an apoplastic tracer (Gong et al., 2006). In this study, after PTS treatment, no significant PTS fluorescence was observed above the background readings in the shoots of either tomato or cucumber (**Figure 3**). This suggests that there was no obvious Si-induced change of apoplastic transport in either tomato or cucumber. Therefore, there was no significant difference in Cd transport via the apoplast in the roots of Cd and Cd + Si treatments in either plant species.

Bars with different letters means significant difference at *P* < 0.05 according to one-way ANOVA (Lower-case and upper-case letters indicate significance for tomato and cucumber, respectively). TBARS, thiobarbituric acid reactive substances.

### MSI, TBARS Content and H_2_O_2_ Content

Compared with the control, Cd toxicity resulted in an obvious decrease of MSI in the roots and leaves of tomato and cucumber seedlings. Si application significantly increased the MSI values under Cd stress, except in cucumber roots (**Table 6**).

**Table 6:** Effect of Si on membrane stability index (MSI) in tomato and cucumber under Cd stress.

	MSI (%)			
	Tomato		Cucumber	
Treatment	Leaf	Root	Leaf	Root
CT	96.3 ± 1.5^a^	73.5 ± 4.5^a^	94.9 ± 1.2^ab^	88.0 ± 2.8^a^
Si	95.5 ± 0.8^a^	78.4 ± 3.7^a^	96.0 ± 0.8^a^	85.2 ± 9.7^ab^
Cd	80.1 ± 1.1^c^	54.7 ± 3.9^b^	85.2 ± 1.4^c^	76.3 ± 3.1^b^
Si + Cd	92.7 ± 0.9^b^	68.6 ± 2.1^a^	94.0 ± 0.8^b^	84.7 ± 4.0^ab^

*Data are means ± SD. Values followed by different letters within a column represent significant difference at P< 0.05 according to one-way ANOVA*.

**FIGURE 4:**
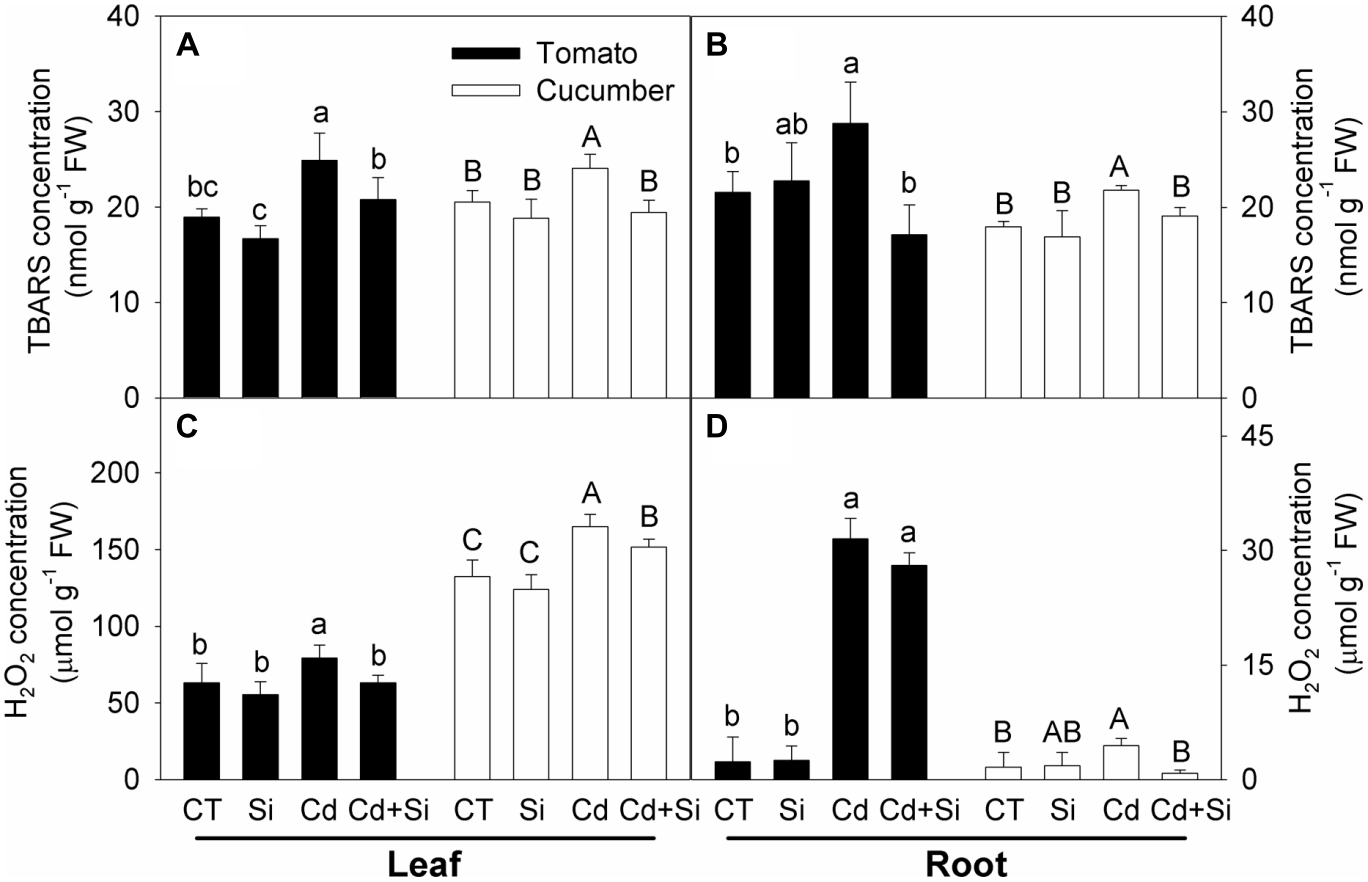
**Effects of Si on TBARS and H_2_O_2_ contents in leaves (A,C) and roots (B,D) in tomato and cucumber under Cd stress**. Bars with different letters means significant difference at *P* < 0.05 according to one-way ANOVA (Lower-case and upper-case letters indicate significance for tomato and cucumber, respectively). TBARS, thiobarbituric acid reactive substances.

Thiobarbituric acid reactive substances contents, which indicate lipid peroxidation, were significantly increased under Cd stress in the leaves and roots of both plants (**Figures 4A,B**), and Si supply significantly decreased the contents (**Figures 4A,B**).

Cadmium stress induced significant increases of H_2_O_2_ concentrations in the leaves and roots of both plants (**Figures 4C,D**). Si addition had no impact on the H_2_O_2_ levels in non-stress conditions. Under Cd stress, added Si significantly decreased the H_2_O_2_ accumulation in tomato leaves and both leaves and roots of cucumber; whereas in tomato roots, Si addition had no significant effect on the H_2_O_2_ accumulation (**Figure 4D**).

### Activities of Antioxidant Enzymes

**FIGURE 5:**
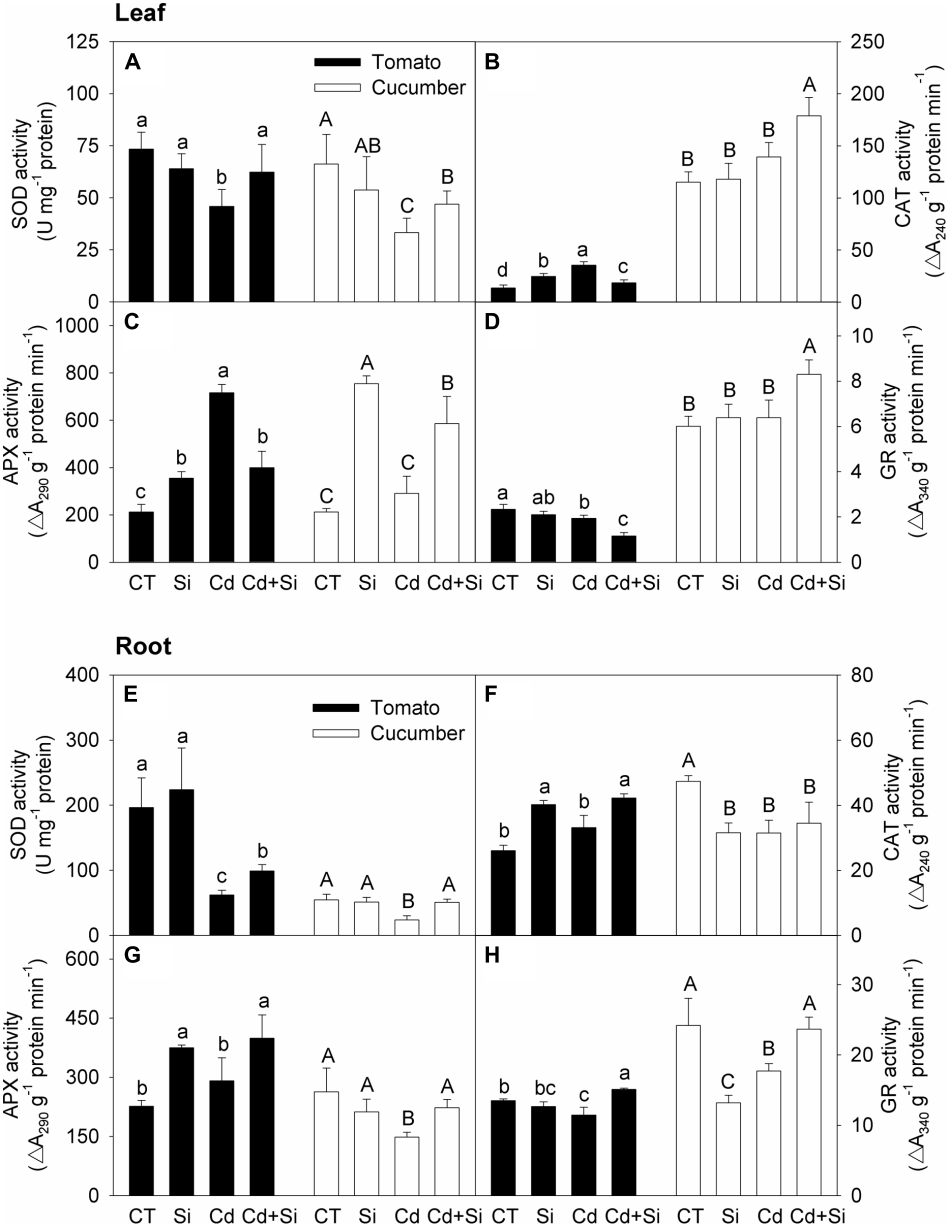
**Effects of Si on the activities of SOD (A,E), CAT (B,F), APX (C,G) and GR (D,H) in leaves (A–D) and roots (E–H) in tomato and cucumber under Cd stress**. Bars with different letters mean significant difference at *P* < 0.05 according to one-way ANOVA (Lower-case and upper-case letters indicate significance for tomato and cucumber, respectively). SOD, superoxide dismutase; CAT, catalase; APX, ascorbate peroxidase; GR, glutathione reductase.

According to **Figures 5A–D**, in tomato leaves, the activities of SOD and GR were depressed while the activities of CAT and APX were increased under Cd stress, and Si supply significantly increased the SOD activity but decreased the CAT, APX, and GR activities in stressed plants (**Figures 5A–D**). As can be seen from **Figures 5E–H**, in tomato roots, the activities of SOD and GR were decreased whereas the activities of CAT and APX were not significantly changed under Cd stress, and Si supply significantly increased the activities of these four enzymes.

As shown in **Figures 5A–D**, in cucumber leaves, the activities of CAT, APX, and GR were not significantly affected by Cd treatment, but the SOD activity was decreased; Si supply increased the activities of the four antioxidant enzymes under Cd stress. As can be seen from **Figures 5E–H**, in cucumber roots, the activities of all four enzymes were depressed under Cd stress, and Si supply significantly enhanced the activities of SOD, APX, and GR but did not affect the CAT activity.

### GSH and AsA Contents

**FIGURE 6:**
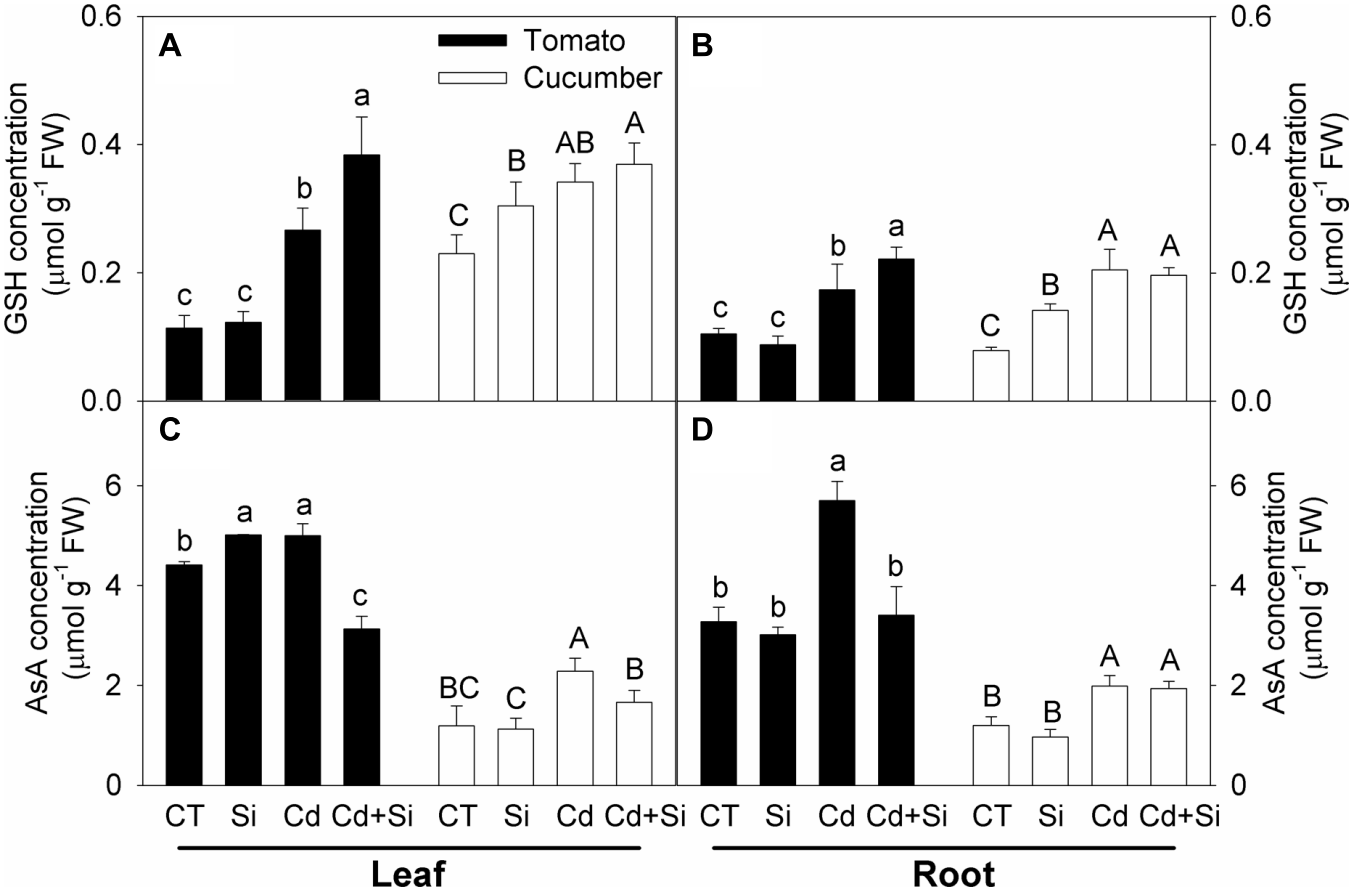
**Effects of Si on GSH (A,B) and AsA (C,D) contents in plants under Cd stress. (A)** GSH content in leaves; **(B)** GSH content in roots; **(C)** AsA content in leaves; **(D)** AsA content in roots. Bars with different letters mean significant difference at *P* < 0.05 according to one-way ANOVA (Lower-case and upper-case letters indicate significance for tomato and cucumber, respectively).

Compared with the control, Cd stress increased the GSH and AsA concentrations in the leaves and roots of both plants (**Figures 6A–D**). Under Cd stress, Si application further increased the GSH concentrations in tomato but did not affect the levels in cucumber (**Figures 6A,B**). By and large, except in cucumber roots, the AsA levels were decreased by Si application in Cd stressed plant tissues (**Figures 6C,D**).

## Discussion

Cadmium contamination in agricultural land is a serious environmental problem. Its uptake by vegetable crops such as cucumber and tomato not only inhibits plant growth but also threatens human health. Therefore, minimizing Cd accumulation in these vegetables is urgent. Si is an element that can be massively accumulated in plants without toxic symptoms. Therefore, application of Si fertilizer may be an ideal means for sustainable development of agriculture. Si-mediated inhibition of Cd accumulation in cereal crops (Wu et al., 2013) also suggests a potential application of Si fertilizer in minimizing Cd pollution in vegetables.

It has been extensively reported that Si supplement can improve plant growth under Cd stress (Liang et al., 2007; Wu et al., 2013). However, there are few reports on the effect of Si on Cd toxicity in low-Si-accumulating plants, such as cucumber and tomato. Our results showed that the deleterious effects of Cd toxicity could be mitigated by Si addition in both species (**Figure 1**). The alleviative effect of Si on plant growth under Cd stress may be related to the reduced Cd accumulation in both plants (**Table 2**). The beneficial Si effect in reducing Cd accumulation in the shoots of both species is very meaningful for vegetable safety and suggests a potential application of Si fertilizer in controlling Cd pollution of these vegetables. Si-induced decrease in shoot Cd accumulation has also been observed in other plants, such as rice (Shi et al., 2005), pakchoi (Song et al., 2009), wheat (Naeem et al., 2014), durum wheat (Rizwan et al., 2012), and maize (Liang et al., 2005). However, Vaculík et al. (2009) observed an increased shoot Cd level by Si application in maize. Lukačová et al. (2013) suggested that the Si effects on shoot Cd accumulation were related to Cd and Si concentrations in maize. These observed differences in silicon effects on shoot Cd accumulation may also be related to plant species. In roots, the effects of Si on Cd accumulation reported in the literatures have also been mixed, showing species- and cultivar-dependence (Song et al., 2009; Vaculík et al., 2009; Shi et al., 2010). Lukačová et al. (2013) suggested that the effect of Si on Cd accumulation in the roots of maize was associated with Cd and Si concentrations: at low Cd and Si concentrations, the root Cd level was increased by added Si, whereas at high Cd and Si concentrations, Si addition decreased the Cd level in roots. Whether this is the case in tomato and cucumber remains to be investigated.

In our experimental conditions, the root-to-shoot Cd translocation was greater in tomato than in cucumber, suggesting a better restriction of Cd transport in cucumber. It is intriguing that compared with the control, Cd toxicity did not significantly affect the root dry weight of cucumber while it significantly decreased the root dry weight of tomato. This suggests that cucumber had better Cd tolerance than tomato based on the present study (**Figure 1**). However, a series of Cd concentrations and different cultivars should be used to further evaluate Cd tolerance difference between tomato and cucumber. Under Cd stress, Si application significantly decreased the root-to-shoot Cd translocation in tomato, whereas it increased the translocation in cucumber (**Table 2**). The reported effects of Si on root-to-shoot Cd translocation have been mixed in previous studies. Zhang et al. (2008) observed that Si supply decreased the root-to-shoot Cd translocation, which was consistent with our results in tomato (**Table 2**). However, some researchers reported Si-enhanced Cd translocation in maize (Vaculík et al., 2009), and *Brassinca juncea* and *B. napus* (Vatehová et al., 2012). In peanut plants, Shi et al. (2010) observed that Si addition decreased root-to-shoot Cd translocation in the sensitive cultivar, but had no effect in the tolerant cultivar. These studies suggest that the effects of Si on Cd translocation may be species-, cultivar-, and Cd-concentration-dependent. In our study, since the Cd concentration in tomato roots was not changed by added Si, the decrease of shoot Cd level is suggested to be attributed to Si-mediated decrease in root-to-shoot translocation. In cucumber plants, however, Si-mediated decrease in shoot Cd level might be attributed to decreased Cd uptake, rather than decreased root-to-shoot transport.

Organic acids have been found to be involved in heavy metal uptake, translocation and accumulation (Kramer et al., 2000; Boominathan and Doran, 2003; Küpper et al., 2004). In the xylem, metallic elements are transported as organic-metal co-complexation (Álvarez-Fernández et al., 2014). Salt et al. (1995) indicated that Cd was mainly coordinated with ligands containing sulfur in roots, and Cd predominately existed as Cd-oxygen or Cd-nitrogen in the xylem sap which probably was Cd-organic acid. Sun et al. (2011) observed that there were positive correlations between Cd concentrations and tartaric or malic acids concentrations in the leaves of *Rorippa globosa* while the Cd concentrations were positively correlated with acetic acid concentrations in the leaves of *R. islandica*. Senden et al. (1995) reported that citric acid pretreatment could increase Cd uptake and translocation in tomato. Han et al. (2006) also observed enhanced Cd uptake and transport by exogenous acetic and malic acids in maize. It can be seen from these studies that organic acids can promote Cd uptake and translocation, with the major organic acid(s) being species-dependant. However, up to date, reports concerning the Si effects on organic acid levels in plants are very limited. Pavlovic et al. (2013) found that Si could alleviate Fe deficiency in cucumber by enhancing the accumulation of Fe-mobilizing compounds (including citric acid and malic acid) in the roots. Bityutskii et al. (2014) also reported that Si supply could effectively alleviate Fe deficiency in cucumber, which was directly related to the increased levels of organic acids (especially citric acid) and phenolic compounds. These studies suggest that Si can regulate the metabolism of organic acids to affect Fe translocation. It will be interesting to investigate whether Si regulates Cd transport by modulating organic acids levels in plants.

In this study, under Cd stress, Si addition did not significantly change the levels of total organic acids in the roots of tomato, and it significantly decreased the levels in the leaves (**Table 4**); in cucumber plants, addition of Si significantly increased the levels of total organic acids in both roots and leaves (**Table 5**). The observed differences of Si-mediated changes in organic acid levels may be related to plant species. In tomato plants, the Si-mediated decrease of total organic acid levels in the leaves with no significant change in the roots might have contributed to the Si-mediated decrease in root-to-shoot Cd translocation (**Table 2**). In cucumber, Si-mediated increases of total organic acid levels in both roots and shoots might have facilitated Cd transport from the roots to the shoots (**Table 2**). To our knowledge, the effect of exogenous Si on organic acid levels under heavy metal stress has seldom been reported. Our results suggest that Si-mediated changes in levels of organic acids may be involved in Cd transport, which may help to elucidate the mechanisms for Si-mediated changes in heavy metal accumulation in the shoots. Further work is needed to elucidate how Si modulates the organic acids metabolism in plants.

Cellular distribution of heavy metal affects its toxicity in plants. The effects of Si on cellular distribution of heavy metal in plants have been investigated by previous researchers with mixed results. Shi et al. (2005) reported that, Si decreased Cd concentrations in rice shoots and roots, and reduced symplastic and apoplastic Cd levels. Under Mn toxicity, Si application decreased the Mn concentration in the symplast by locating more Mn into the apoplast (Rogalla and Römheld, 2002; Dragisic Maksimovic et al., 2012). In our study, Si supply decreased Cd concentrations in the symplast of leaves and roots in both plants (**Table 3**), which might have contributed to decreased oxidative stress under Cd stress as discussed below. However, the Si effects on Cd concentrations in the apoplast were different in the two species. The proportions of apoplastic Cd level in the roots and leaves were increased by added Si in tomato, but decreased by added Si in cucumber (**Table 3**). These changes were negatively related to the changes of Cd TFs, suggesting that the cell wall components might also affect Cd-binding capability, therefore root-to-shoot translocation. These studies also suggest that the effect of Si on cellular distribution of heavy metal is species-dependent.

Cell walls play pivotal roles in regulating plant growth and defense strategies (Krzeslowska, 2011). Chen et al. (2013) reported that, under Cd toxicity, the contents of pectin and cellulose were positively correlated with Cd absorption, whereas hemicellulose content was negatively associated with Cd uptake. However, little information is available for Si-mediated alleviation of Cd toxicity in relation to cell wall components. In our study, under Cd stress, the spectrum peak intensities were dramatically decreased in tomato roots, while the decreases were much less in cucumber roots (**Figure 2**). These results are consistent with the changes of root dry weights under Cd stress and also suggest a better Cd tolerance of cucumber (**Figure 1**). Under Cd toxicity, Si addition increased the absorbance values of spectra in both species, especially between 900 and 1300 cm^-1^ where included cellulose (1155 cm^-1^), hemicellulose (1068 cm^-1^), and pectin (1105 cm^-1^) (**Figure 2**). The increase of root polysaccharide contents by Si addition in tomato was consistent with the increase in the proportion of apoplastic Cd (**Table 3**). However, a similar relationship did not exist in cucumber roots. In consideration of the complicated relationships between different polysaccharides and heavy metal uptake (Zheng et al., 2004; Yang et al., 2011; Chen et al., 2013), there is a possibility that the polysaccharide components and proportions in the two plants were different, and the polysaccharide components of Si-enhanced synthesis were also different. However, in view of the observed differences in the roles of different polysaccharides or even the same polysaccharide by different researchers in Cd translocation (Zheng et al., 2004; Yang et al., 2011; Chen et al., 2013), more work is needed to clarify these speculations in future.

In rice, Si was mainly deposited in the vicinity of the endodermis, and the deposition partially blocked apoplastic Cd transport and therefore decreased shoot Cd concentration (Shi et al., 2005). In our study, PTS – an apoplastic tracer, was used to determine the apoplastic transport of Cd. However, there was no obvious difference in Cd transport via apoplastic pathway with or without added Si in either cucumber or tomato (**Figure 3**). These results suggest that, unlike rice, Si-deposition-induced physical blockage did not contribute to Si-mediated decrease in shoot Cd accumulation in either cucumber or tomato – two less-Si-accumulating plants.

Cadmium toxicity induced oxidative damage could be alleviated by enzymatic and non-enzymatic antioxidants in plants (Gonçalves et al., 2007). It is also widely accepted that Si application can ameliorate oxidative stress through regulating antioxidant enzyme activities and non-enzymatic antioxidant substance levels in plants (Liang et al., 2007; Wu et al., 2013). Song et al. (2009) reported that Si supply reduced the malonaldehyde and H_2_O_2_ contents under Cd stress by enhancing antioxidant enzyme activities and non-enzymatic antioxidant contents in pakchoi. Liu et al. (2013b) reported that Cd-toxicity-induced accumulations of malonaldehyde and H_2_O_2_ were decreased by added Si, and the activities of antioxidant enzymes were also decreased. However, the possible role of antioxidant defense in Si-mediated alleviation of Cd toxicity in either tomato or cucumber has not been systematically investigated. In our work, Si supplement significantly mitigated lipid peroxidation and increased the MSI in leaves and roots of both tomato and cucumber seedlings (**Figure 4**, **Table 6**). These findings are consistent with previous studies (Song et al., 2009; Liu et al., 2013b). However, the changes in enzymatic and non-enzymatic constituents were different between cucumber and tomato. In cucumber, the H_2_O_2_ contents were decreased by added Si in leaves and roots under Cd treatment (**Figures 4C,D**), which might be related to enhanced antioxidant enzymes activities (**Figure 5**). In tomato leaves, Si application increased the SOD activity but decreased the activities of other antioxidant enzymes under Cd stress (**Figure 5**), indicating that the plants did not need high activities of antioxidant enzymes to scavenge excessive ROS in leaves because the H_2_O_2_ content in Si + Cd treatment was at the control level (**Figure 4C**). However, in tomato roots, the H_2_O_2_ contents were similar with or without added Si under Cd toxicity (**Figure 4D**), and Si-mediated increases in antioxidant enzyme activities (**Figure 5**) and GSH concentration (**Figure 6B**) might be an adaptive response to counteract the stress. In consideration of the different responses of antioxidant enzymes activities in different tissues and species, it seems that Si modulated the antioxidant defense system as a secondary response. However, further work is needed to clarify this.

## Conclusion

Silicon could alleviate Cd-induced growth inhibition in tomato and cucumber. The alleviation was related to the decreased Cd accumulation in the shoot. In tomato, the decreased Cd concentration is suggested to be attributed to Si-mediated decrease in root-to-shoot translocation; whereas in cucumber, Si-mediated decrease in shoot Cd level might be attributed to decreased Cd uptake by roots. Si could modulate the organic acid levels and cell wall components, which might be involved in Cd transport regulation in both plants. Si-mediated alleviation of oxidative damage also contributed to Cd tolerance in both species.

## Conflict of Interest Statement

The authors declare that the research was conducted in the absence of any commercial or financial relationships that could be construed as a potential conflict of interest.
